# Activity dependent dissociation of the Homer1 interactome

**DOI:** 10.1038/s41598-022-07179-3

**Published:** 2022-02-25

**Authors:** Mason Stillman, Jonathan D. Lautz, Richard S. Johnson, Michael J. MacCoss, Stephen E. P. Smith

**Affiliations:** 1 Center for Integrative Brain Research, Seattle Children’s Research Institute, Seattle, WA USA; 2 Department of Genome Sciences, University of Washington, Seattle, WA USA; 3 Department of Pediatrics, University of Washington, Seattle, WA USA; 4 Graduate Program in Neuroscience, University of Washington, Seattle, WA USA; 5 Present Address: Dartmouth-Hitchcock Medical Center Psychiatry Residency Program, Dartmouth, NH USA

**Keywords:** Molecular neuroscience, Synaptic plasticity, Mass spectrometry

## Abstract

Neurons encode information by rapidly modifying synaptic protein complexes, which changes the strength of specific synaptic connections. Homer1 is abundantly expressed at glutamatergic synapses, and is known to alter its binding to metabotropic glutamate receptor 5 (mGlu5) in response to synaptic activity. However, Homer participates in many additional known interactions whose activity-dependence is unclear. Here, we used co-immunoprecipitation and label-free quantitative mass spectrometry to characterize activity-dependent interactions in the cerebral cortex of wildtype and Homer1 knockout mice. We identified a small, high-confidence protein network consisting of mGlu5, Shank2 and 3, and Homer1–3, of which only mGlu5 and Shank3 were significantly reduced following neuronal depolarization. We identified several other proteins that reduced their co-association in an activity-dependent manner, likely mediated by Shank proteins. We conclude that Homer1 dissociates from mGlu5 and Shank3 following depolarization, but our data suggest that direct Homer1 interactions in the cortex may be more limited than expected.

## Introduction

The Homer protein family is encoded in mammals by three evolutionarily conserved genes, and is expressed widely throughout the brain where it is enriched in post-synaptic density preparations^1,2^. The basic protein structure of Homer proteins consists of an EVH1 ‘head’ domain, which serves as a ligand binding site for the PPXXF motif expressed on a variety of proteins, and a long coiled-coil “tail”, which tetramerizes with other Homer proteins to produce an X-shaped complex with four exposed EVH1 ‘heads’^3^. Confirmed binding partners include metabotropic glutamate receptor 5 (mGlu5)^1^, IP3 receptors^4^, several actin-binding proteins^5,6^, and the Shank family of proteins^7^. Shanks bind numerous postsynaptic proteins including NMDARs, AMPARs, scaffold proteins, and signaling effectors, functionally linking these proteins with Homers in large macromolecular protein complexes^8^. Moreover, due to the Shank protein family’s ability to homo-dimerize, 1:1 mixtures of Shank and Homer form a repetitive mesh structure in vitro, resembling a chain-link fence^3^. By extension, in vivo, a Shank–Homer scaffold may form an extensive synapse-wide network below the PSD in an area termed the “PSD pallidum”^9^. While the extent of multimerization in vivo has not been demonstrated directly, the fact that each synapse contains approximately 300–350 copies each of Homer and Shank proteins, a 1:1 ratio^10^, make a synapse-spanning molecular mesh composed of Shank and Homer proteins a distinct possibility.

One might expect that a protein that plays such an important scaffolding role at the synapse would be tightly regulated. In fact, the binding of the Homer EVH1 domain to its targets is regulated by both post-translational and translation-dependent mechanisms. First, synaptic activity induces the CamKII-mediated phosphorylation of SER117 (on Homer1), which reduces the binding of Homer1 to mGlu5^11^, and by homology, presumably to other targets as well. Secondly, synaptic activity also induces the translation of a short isoform of Homer1, called Homer1a, which lacks the c-terminal CC domain and thus the ability to form multimers with other Homer proteins^12^. Homer1A binds in a dominant negative manner to Homer binding partners, freeing them from the static long-form-Homer scaffold (for review, see^13^). Thus, in response to intense synaptic activity, or during sleep^14^, Homer binding partners will be rapidly released from the scaffold via phosphorylation, then maintained in the released position by binding of the newly translated dominant negative form.

Release from the Homer scaffold can have profound effects on the activity of the Homer binding partner. By binding to the Shank–Homer scaffold, mGlu5 is spatially segregated from NMDARs, which is important for downstream signaling through ERK and mTOR pathways^15^. When mGlu5 releases from the Homer scaffold, it physically associates with NMDARs, and prevents NMDAR-mediated activation of these pathways^15–17^. This may shut synapses “off” following activity, so that plasticity in response to a strong input can proceed without interference from subsequent incoming stimuli. In Shank3 KO mice, the regulation of NMDA signaling and synaptic plasticity is disrupted, and can be rescued by the expression of an artificial scaffold linking mGlu5 to the PSD^15^. Similarly, in Fragile X syndrome, mGlu5-Homer disruption seems play an active role in disease pathogenesis^11,18,19^.

Our group previously used an antibody-based Quantitative Multiplex co-Immunoprecipitation (QMI) approach to measure activity-dependent changes in synaptic protein–protein interaction networks^20–22^. We simultaneously measured ~ 400 binary co-associations among a network of 20 synaptic proteins following treatment of cells or acute brain slices with NMDA, glutamate or KCl. While we did observe some increases in co-associations among a few proteins (e.g. AMPAR_PSD95), the vast majority of activity-dependent changes in protein interactions were dissociations. Moreover, Homer and Shank proteins were the hubs of this network dissociation, accounting for more binary changes than any other measured protein. Our data inspired us look more closely at activity-dependent Homer1 interactions in cortical neurons, since the protein interactome is notoriously under-characterized^23^, especially when it comes to activity-dependent interactions in specific tissue types^24^. In fact, a prior study of Homer2 interactions in mouse brain tissue identified 15 novel interactions, while only recapitulating 3/31 interactions found in databases^25^. Here, we used Homer1 co-immunoprecipitation followed by label-free quantitative mass spectrometry to characterize the activity-dependent interactions of Homer1. We found that Homer1 can be unambiguously associated only with Shank, Homer and mGlu5 proteins. When we expanded our search to include proteins whose abundance was reduced following neuronal depolarization, we identified several other potential interaction partners. However, our analysis suggests that these proteins are in a shared complex with Homer via a Shank intermediate, and do not directly interact with Homer1. Overall, our results suggest a surprising specificity of Homer1 interactions in the cortex, limited to Homer proteins, Shank proteins, and mGlu5. We speculate that the importance of mGlu5 regulation may have led to the specialization of a highly specific and tightly regulated activity-dependent scaffolding system. However, several limitations involving protein solubility, potential antibody cross-reactivity with other Homer species, or mass spectrometer detection that may have prevented detection of bona fide interactors are discussed in the limitations section, so these results should be interpreted with caution.

## Results

**Figure 1:**
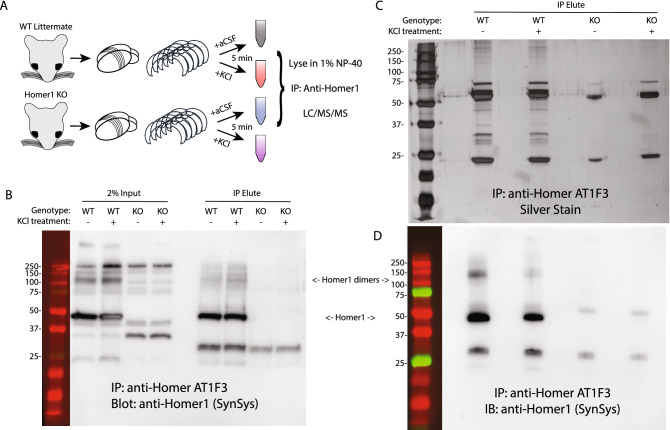
Sample preparation for mass spectrometry. (**A**) Experimental design. (**B**) Western blot of representative immunoprecipitation showing Homer1 bands at the expected molecular weight. (**C**) Silver stain showing protein co-IP’d in WT and KO mice. (**D**) Western blot on the same blot as the silver stain showing Homer1 protein at the expected molecular weight.

We performed co-IP followed by quantitative mass spectrometry to characterize the full complement of activity dependent Homer1 interactions in mouse cortex. We cut acute brain slices from 4 WT and 5 Homer1 knockout (KO) mice using the protective recovery method^26^ to maintain the slices alive ex vivo, and stimulated them for 5 min with either control artificial cerebro-spinal fluid (aCSF) or 50 mM KCl, which induces depolarization of neurons, calcium-mediated activation of signaling pathways, and dissociation of Homer1 from mGlu5^20,22^. We solubilized cortices in 1% NP-40 detergent^27^ and immunoprecipitated Homer1 with the mouse monoclonal antibody clone AT1F3, raised against full-length human Homer1 (Fig. 1A). We validated successful IP by western blot and silver stain, and confirmed the lack of Homer1 in the KO lysate and IP eluate (Fig. 1B–D). We then quantified the co-associated proteins by label-free quantitative liquid chromatography-tandem mass spectrometry followed by MS1 full-scan filtering in data-independent acquisition mode.

**Figure 2:**
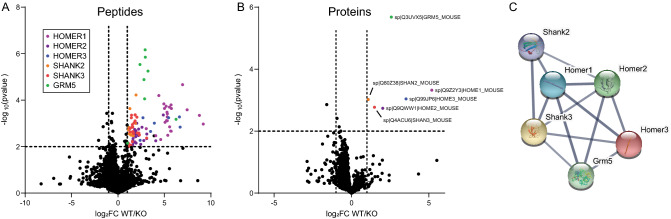
Co-IP’d peptides and proteins identified comparing WT vs KO in the aCSF condition. (**A**) Volcano plot showing peptides that were enriched in WT vs KO cortex. Key shows the color-coding of the peptides. (**B**) Volcano plot showing proteins that were enriched in WT vs KO cortex. Enriched proteins are labeled, and colors are consistent with (**A**). (**C**) StringDB node-edge diagram of the identified proteins highlighting the extensive known interactions.

We identified 9560 unique peptides, and first focused on identifying Homer1-binding proteins by identifying ‘peptides of interest’ that were significantly enriched in WT-ACSF compared to KO-ACSF with at least a log_2_FoldChange of 1 and an FDR of 1% (Fig. 2A), yielding 101 significantly enriched peptides derived from only 7 unique proteins. Homer1 was associated with the largest number of significant peptides (27), followed by Shank3 (25), Homer2 (23), Shank2 (12), Homer3 (8), and mGlu5 (8) (Table 1). Homer proteins are known to tetramerize with other Homer family members via their c-terminal coiled-coil domain, so the co-immunoprecipitation of Homer2 and 3 was expected. Shank2 and 3 and mGlu5 are known to interact with Homers1–3 via the Homer EVH1 domain binding to conserved Pro-Pro-x-x-Phe motifs. A single significant peptide from the protein JOS1, a deubiquitinating enzyme with no known Homer binding motifs, was dismissed as likely noise. It was surprising that although 24 peptides from Shank1 were detected, none met our criteria for statistical significance; in fact, only 4 had a − log_10_(value) > 1, and only 3 had a log_2_FC > 1.

**Table 1:** Homer-interacting proteins identified by mass spectrometry.

Protein name	Number of peptide significant for WT vs KO	Total number of peptides detected	Uniprot name	Protein name
Homer1	27	32	HOME1	Homer protein homolog 1
Shank3	25	39	SHAN3	SH3 and multiple ankyrin repeat domains protein 3
Homer2	23	28	HOME2	Homer protein homolog 2
Shank2	12	31	SHAN2	SH3 and multiple ankyrin repeat domains protein 2
Homer3	8	10	HOME3	Homer protein homolog 3
mGluR5	8	8	GRM5	Metabotropic glutamate receptor 5
JOS1	1	1	JOS1	Josephin-1

We next combined individual peptide data across proteins, and identified 1436 unique proteins (971 with more than 1 peptide detected). Of these, 6 proteins were significantly enriched in WT mice: Homer1–3, Shank2–3 and mGlu5 (Fig. 2B). Overall, these proteins are known to interact extensively with each other, with high confidence scores in StingDB^28^ (Fig. 2C). Notably, Shank1 was 613th on the list, with a log2FC = 0.38 and − log(pvalue) = 0.31. Compared to the large number of reported interactions for Homer1 in protein interaction databases, we identified a relatively low number of significant hits in our stringent WT-to-KO comparison. We searched our list for proteins listed as Homer1 interactors in BioGRID or UniPROT, and identified 15 additional proteins out of 69 total. These 15 proteins, while detected, were not significantly enriched (Table S1), which argues against these proteins interacting with Homer1 in the adult mouse cortex.

**Figure 3:**
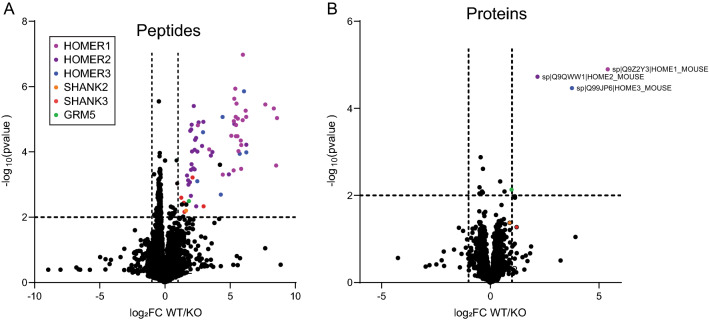
Co-IP’d peptides and proteins identified comparing WT vs KO in the KCl condition. (**A**) Volcano plot showing peptides that were enriched in WT vs KO cortex. Key shows the color-coding of the peptides. (**B**) Volcano plot showing proteins that were enriched in WT vs KO cortex. Enriched proteins are labeled. Comparing to Fig. 2, the loss of GRM5 (green) and Shank2,3 proteins (orange/red) is apparent.

We next turned our attention to the activity-dependence of these interactions. We^20,21^ and others^11,15^ have previously reported that Homer1 dissociates from mGlu5 (and other interactors) following KCl treatment of cultured neurons or acute cortical slices stimulated in vitro. We compared peptides IP’d from WT-KCL treated mouse brain slices with those IP’d from KO-KCL-treated slices using identical methods as above. Of 9560 unique peptides, 71 peptides had at least a log_2_FoldChange of 1 and p < 0.01 (Fig. 3A), derived from 10 unique proteins; however, only four proteins had more than a single peptide detected: Homer1, Homer2, Homer3 and Shank3. After binning peptide data into proteins, only three proteins met the criteria of least a log_2_FoldChange of 1 and p < 0.01: Homer 1, 2 and 3 (Fig. 3B). mGlu5, Shank2 and Shank3 were no longer significantly enriched, indicating that they released from Homer1 following KCl depolarization (although mGlu5 was close to criteria with log2FC = 0.976).

**Figure 4:**
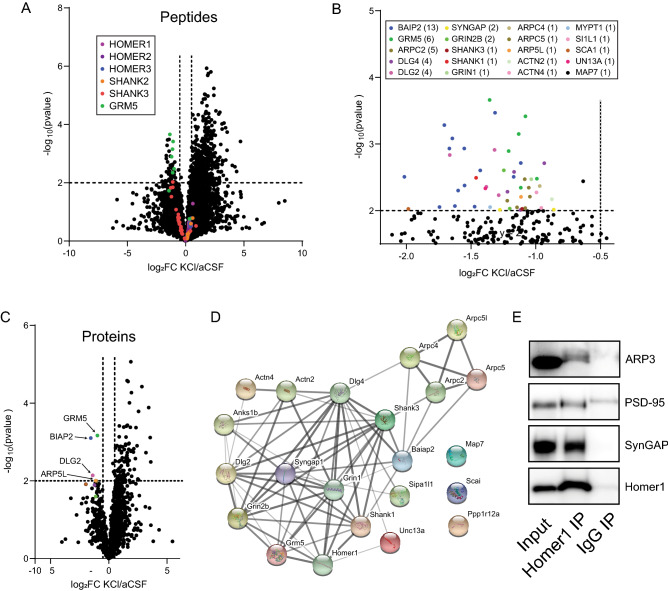
Co-IP’d peptides and proteins identified comparing aCSF vs. KCl in WT lysates. (**A**) Volcano plot showing peptides that were enriched and depleted in aCSF vs. KCl. Peptides that were identified as enriched in WT vs KO in the aCSF condition are color-coded as shown in the key. (**B**) The same volcano lot from (**A**) is shown with the scale changed to highlight significantly depleted peptides, and all peptides that meet significance criteria color-coded by protein identity. Key shows color-coding of peptides by protein identify. (**C**) Volcano plot showing proteins that were depleted in aCSF vs. KCl. Depleted proteins are labeled. (**D**) StringDB node-edge diagram of the identified peptides from (**B**), highlighting the known interactions. (**E**) IP-western blots confirm that ARP3, PSD95 (DLG4) and SynGAP co-associate with Homer1, but not with nonspecific IgG control.

We next took a different approach to identifying proteins that dissociated from Homer1 following KCl stimulation, by comparing peptides detected in the WT aCSF vs. WT KCl conditions. We first focused on the 101 peptides that were significantly enriched in the WT-aCSF vs KO-aCSF comparison. We found that the abundance of peptides associated with Homer1 slightly but non-significantly *increased* following KCL stimulation, as did peptides from Homer2, Homer3, and Shank2. Conversely, one peptide associated with Shank3, and 6 associated with mGlu5 reached the threshold of p < 0.01, log2FC > − 0.5, which we used to define significantly changed peptides; other Shank3 and mGlu5 peptides trended towards significance (colored dots in Fig. 4A). These data further support the activity-dependent dissociation of Homer from mGlu5 and Shank3, but not from Shank2 or other Homers.

We next looked at all peptides that were significantly downregulated following KCl, without regard to weather they were enriched in the WT vs KO. We identified 43 peptides derived from 20 proteins (Fig. 4B). After binning peptide data by protein, only 4 proteins met our criteria for significance: mGlu5, BIAP2, DLG2, and ARP5L (Fig. 4C). Even though most of the peptides and proteins were not identified in the original WT vs KO comparisons, we visualized previously-documented interactions among the identified peptides/proteins using the String database, which revealed a highly interconnected network (Fig. 4D) with a PPI enrichment p-value of 10^–16^. The top 3 GO Molecular function enriched terms in this network were “protein-containing complex binding” (p = 7.7e^−10^), “glutamate receptor binding” (p = 1.13e^−9^) and “structural constituent of the postsynapse” (p = 8.37e^−8^), while the top KEGG pathways were “glutamatergic synapse” (p = 7.56e^−9^) and “regulation of actin cytoskeleton” (p = 2.94e^−7^). These data are consistent with previously reported functions of Homer1 as a synaptic scaffold that links synaptic activity (e.g. GRIN1, GRIN2B, MGLU5) to changes in actin polymerization (e.g. ACTN2, ACTN4, ARPC2,4,5,5L)^5,7,11^. These features of the dataset increased our confidence that the identified proteins occur in large macromolecular complex(es) with Homer1, possibly linked via Shank intermediates. To confirm that these proteins complex with Homer1, we performed co-IP western blots of mouse cortical lysate for 4 targets identified as changed by KCL in WT samples: Actinin4, the ARP complex, DLG4 (PSD95), and SynGAP (Fig. 4E). Using nonspecific IgG as an IP control, we confirmed that Homer1 co-IPs with each of these protein targets. Additionally, we have previously demonstrated that the interaction between Homer1 and DLG4 or SynGAP dissociate following KCl treatment^20^. One additional target, SCAI, showed non-specific IgG binding as well as Homer1 binding and so was not confirmed.

In addition, 564 peptides derived from 176 proteins were significantly enriched following KCl treatment. Binned by protein, 96 proteins reached the threshold for significance. In both cases, the most significant protein was VATA, and the peptide/protein with the largest fold-change was BRSK2/ATX2, respectively. However, not a single KCl-enriched peptide or protein was identified in any of the WT vs KO comparisons. Enriched GO terms (based on peptide-level data) included “purine ribonucleotide binding” (p = 7.7e^−22^), “anion binding” (p = 2.1e^−21^), and “protein binding” (p = 2.8e^−21^), while KEGG pathways included “synaptic vesicle cycle” (p = 2.1e^−13^), and “endocytosis” (p = 1.08e^−10^). Given that no proteins identified are known Homer1 interactors, that the GO terms suggest non-specific binding, and that none of these putative interactors were identified in WT vs KO comparisons, we suspect that these proteins represent changes in non-specific binding following KCl treatment. However, we include the full dataset in supplementary material for further reference (Tables S2, S3).

## Discussion

Homer proteins, in partnership with Shanks, are thought to form a scaffold that coordinates the activity of glutamate receptors and orchestrates synaptic plasticity events^13^. Our prior data suggested that Homer played a central role in regulation of synaptic activity, but was limited by our antibody-based, candidate-based approach^20,21^. Here, we used data-independent acquisition mass spectrometry with the goal of cataloguing the full repertoire of Homer1 dissociations following synaptic activity. We confirmed that Homer1 interacts with Homer 2 and 3, as well as Shank 2, 3 and mGlu5. These interactions were already well known, and already present in databases such as BioGRID. While these results are not novel, they add confidence that these known interactions occur in mouse brain.

A second goal of this study was to explore the activity-dependent dissociation of Homer1 from interaction partners beyond the well-documented case of mGlu5. We showed evidence of this dissociation in two ways: first, following KCl treatment, mGlu5, Shank2 and Shank3 were no longer significantly enriched in a WT vs. KO comparison. Second, comparing WT aCSF vs WT KCl-treated slices, we observed a significant depletion of 6/8 peptides derived from mGlu5, and a trend towards significant depletion of Shank3, although only a single peptide reached the threshold of statistical significance. Interestingly, although Shank2 was no longer significantly enriched in WT vs. KO following KCl, Shank2 peptides did not show a clear trend towards depletion in comparisons of WT aCSF vs. WT KCl, which may hint at differential regulation of Shank isoforms. A previous study using immuno-EM found less mobility of Shank2 vs. Shank1 following KCl depolarization^29^. Differential activity-dependent regulation of Homer-Shank isoforms may deserve further study.

In comparisons between WT aCSF and WT KCl-treated slices, we also detected a significant reduction (at the protein level) in the co-association of DLG2 (SAP97) and BAIAP2, with a trend towards significance for DLG4 (PSD95), ARPC2 and ARPC5. At the peptide level, we observed a significant reduction in multiple peptides derived from the aforementioned proteins, as well as from NMDAR2B and SynGAP. Single peptides derived from several ARP complex proteins and NMDAR1 were also depleted. Since these proteins were not identified in WT vs KO comparisons, they must be interpreted with caution. However, given what is known about the structure of the PSD, a compelling model can be proposed: DLG2 and 4, NMDARs and SYNGAP do not possess canonical Homer binding motifs, but are known to bind with Shanks via the intermediates DLGP1–4^9,30^. It should be noted, however, that DLGP1–4 proteins were identified in our data, but none decreased with KCl treatment- in fact many showed a non-significant trend towards increasing. Nevertheless, a complex containing Shank-PSD95-NMDAR is well established^8,9^. The dissociation of Shanks from Homer1 would be a concise explanation for the coordinated dissociation of Homer1, Shank3, NMDARs, DLGs and SynGAP observed here, and in our previous studies^20–22^.

Our data also suggest that the ARP complex, an actin assembly complex containing ARPC2, 4 and 5, and ARP5L, dissociates from Homer following activity. One possible linkage between Homer and the ARP complexes could involve Drebrin, an actin-bundling protein that can bind to Homer via an EVH1 domain-to-PXXPF motif interaction^5^. However, although we detected 33 Drebrin peptides in our samples, none was enriched in WT vs. KO, nor depleted in KCl vs. aCSF, suggesting Drebrin may not bind to Homer1 in mouse cortex. An alternative explanation for ARP-Homer co-association would be through Shank proteins, which are known to interact with cortactin^31^ (UniProt ID: SRC8), an actin-nucleating protein that recruits and activates the ARP complex^32,33^. Indeed, 8/8 SRC8 peptides that we identified showed (non-significant) reductions with log2FC between − 0.5 and − 0.8 following KCl treatment, and the SRC8 protein overall showed a 0.68 Log2FC and a p-value of 0.07, similar to ARPC3. It should be noted, however, that SRC8 was not enriched in WT vs KO comparisons at either the peptide or protein level. These data suggest that following synaptic activity, while Shank is recruiting actin into spines via Cortactin and the ARP complex^31^, Homer is simultaneously dissociating from the Shank scaffold.

The final protein that we identified as dissociating from Homer1 is BAIAP2, also known as IRSp53. BAIAP2 knockouts show NMDAR hyperactivity and social and cognitive deficits, and the gene has been linked to autism, schizophrenia and ADHD in humans^34^. BAIAP2 is known to co-associate with DLG4^35^, Shank1^36^ and Shank3^37^, and plays a role in linking CDC42 to the synapse and regulating actin dynamics^34^. BAIAP2 has also been reported to coimmunoprecipitate with Homer2^25^, although given that it lacks a known Homer-binding motif, the most parsimonious explanation for the co-association may be indirect via Shank.

### Limitations

We were surprised at the low number of interactions identified, which could be due to limitations of our approach. One potential source reason for the low number of interactors identified could be cross-reactivity of our Homer1 antibody with Homer 2 or 3. Out of 74 Homer1 interaction partners listed in UniPROT and BioGRID, our study confirmed 5 proteins and detected peptides from an additional 15 previously identified interactors that were not enriched in WT vs KO. If our antibody bound significant amounts of Homer 2 or 3, this might raise the amount of these 15 targets in the KO sample, precluding their significance in the WT vs KO comparison. We showed a lack of Homer1 immunoreactivity in KO samples, and an enrichment of Homer 2 and 3 in WT vs KO animals, but we cannot exclude the possibility that low levels of antibody binding to Homer 2 and 3 in the KO animal contaminated our results. Future experiments could use additional anti-Homer antibodies, or Homer 1,2,3 triple-knockout mice, to further query these 15 targets.

However, an additional 54 previously identified interactors were not detected in any samples. This may be due to cell type; while experiments in BioGRID include yeast 2-hybrid screens and experiments performed in immortalized cultured cells, we used primary cortical tissue where these proteins may not be expressed. Alternatively, our detergent selection could have prevented us from sufficiently solubilizing Homer and its interactors. In fact, we have previously demonstrated that that detergent has a profound effect of synaptic protein–protein interactions detected by co-immunoprecipitation^27^. Here, we used NP-40 because our previous work found that it preserved the largest number of Homer interactions on our QMI antibody panel; however, it only solubilizes ~ 50% of total Homer protein^27^. While a stronger detergent such as deoxycholate could completely solubilize Homer, it also dissociated the very interactions that we are attempting to detect, measured by co-IP or size exclusion chromatorgraphy^27^. Future experiments could explore different detergents, or use in vivo labeling methods to identify additional potential Homer interactors. Similarly, perhaps if we were to enrich for synaptic material using synaptosomal or PSD preparations, we would have identified more interactors. PSD preparations are problematic because they require solubilization with harsh detergents and the associated loss of protein interactions, but synaptosome or synaptodendrosome preparations may yield additional synaptic Homer interactors. While it is possible that the caveats discussed above precluded our detection of additional Homer interacting partners, our data suggest that Homer may be quite specific with its interaction partners in cortex, largely limiting its binding to Homers, Shanks, and mGlu5.

## Conclusions

Overall, the small number of co-associating proteins that we detected in mouse cortical lysates was unexpected. While this could be due to our IP conditions or to technical limitations of our mass spectrometer, the fact that we detected 15 previously reported proteins but did not find enrichment in WT vs. KO, and the fact that we did detect abundant Homer, Shank and mGlu5 proteins, argues against a technical failure. Rather, our data suggest that in the mouse cortex, Homer may perform a single, rather specific function of activity-dependent regulation of a linkage between Shanks and mGlu5. While it is somewhat surprising that a protein with such extensive activity-dependent regulation and such a large repertoire of reported interactions would be relegated to a single functional role in mouse cortex, this may reflect the importance of mGlu5 regulation at the excitatory synapse.

## Materials and methods

### Animals

Homer1^tm1Mhd^ (stock: 023313) mice were originally obtained from The Jackson Laboratory (Bar Harbor, Maine) and maintained in an in-house breeding colony and maintained on a background of C57BL/6J (stock: 000664). All mice were separated by sex, and housed with littermates, with no more than five mice/cage. Food and water were provided ad libitum. Male KO mice were single-housed due to hyper-aggression. The experiments were approved by the Seattle Children’s Research Institute Animal Care and Use Committee. All methods were carried out in accordance with relevant guidelines and regulations and are reported in accordance with ARRIVE guidelines.

### Slice preparation

Mice were deeply anesthetized with Isoflurane and transcardially perfused with NMDG protective recovery solution (93 mM NMDG, 2.5 mM KCl, 1.2 mM NaH_2_PO_4_, 30 mM NaHCO3, 20 mM HEPES, 25 mM glucose, 2 mM thiourea, 5 mM Na-ascorbate, 3 mM Na-pyruvate, 0.5 mM CaCl_2_·4H_2_O, and 10 mM MgSO_4_·7H_2_O; titrated to pH 7.4 with concentrated hydrochloric acid and bubbled with 5%CO_2_ in O_2_). Brains were quickly removed and coronal cortical slices were sectioned at 400 µm thickness using a vibratome. Slices were immediately hemisected with a sharp razor blade and each half placed in an alternate treatment group, with treatment groups being arbitrarily assigned. Slices were initially recovered in NMDG protective recovery solution for 15 min at 32 °C, then transferred to a modified HEPES holding solution [92 mM NaCl, 2.5 mM KCl, 1.2 mM NaH_2_PO_4_, 30 mM NaHCO_3_, 20 mM HEPES, 25 mM glucose, 2 mM thiourea, 5 mM Na-ascorbate, 3 mM Na-pyruvate, 2 mM CaCl_2_·4H_2_O, and 2 mM MgSO_4_·7H_2_O; pH 7.4, bubbled with 5% CO_2_ in O_2_] for an additional 90 min recovery at room temperature.

### Slice stimulation and lysate preparation

Control (aCSF) slices were transferred to aCSF [129 mM NaCl/5 mM KCl/2 mM CaCl_2_/1 mM MgCl_2_/30 mM Glucose/25 mM HEPES; pH 7.4] and KCl slices were transferred to high K + aCSF [84 mM NaCl/50 mM KCl/2 mM CaCl_2_/1 mM MgCl_2_/30 mM Glucose/25 mM HEPES; pH 7.4] for 5 min at 37 °C. Following stimulation, slices were transferred to a glass/Teflon homogenizer with 300 μl ice cold 1% NP-40 lysis buffer [150 mM NaCl, 50 mM Tris (pH 7.4), 1% NP-40, 10 mM sodium fluoride (Sigma, 201154), 2 mM sodium orthovanadate (Sigma, 450243) + Protease/phosphatase inhibitor cocktails (Sigma, P8340/P5726)], using 12 strokes of a glass‐teflon homogenizer. Lysates were incubated for 15 min on ice, and cleared by centrifugation at high speed in a benchtop refrigerated centrifuge.

### Immunoprecipitation

Protein concentration was measured by BCA assay (Pierce) and normalized by the addition of lysis buffer. Lysates were pre-cleared by incubation with 25 μl of Protein G magnetic beads (NEB) for 1 h at 4 °C with rotation followed by magnetic removal of beads. 10 μg of anti-Homer1 antibody (AT1F3, LSBio Cat# C103482) was incubated overnight at 4 °C with rotation, then 25 μl of Protein G beads were added for 2 h at 4 °C with rotation. Lysate was removed and beads were washed two times with wash buffer (50 mM tris (pH 7.4), 100 mM NaCl, 1% bovine serum albumin, and 0.02% sodium azide), then two additional times with TBS (100 mM NaCl, 50 mM Tris, pH 7.4). Proteins were eluted twice with 50ul of 0.2 M Glycine (pH 2.5) incubated for 5 min. The two eluates were combined, and pH was neutralized with 20 μl of 1.0 M Tris–HCl (pH 9.0). Proteins were then precipitated using methanol/chloroform^38^ and dried on a vacuum centrifuge.

### Western blotting

For western blots, proteins (20 μg per lane) were separated by SDS-PAGE. For silver staining, blots were developed with the Pierce Silver Stain for Mass Spectrometry Kit (Pierce Cat# 24600). For western blotting, proteins were transferred to a PVDF membrane (Millipore), blocked in 4% milk in TBST (0.05 M Tris pH7.2, 0.15 M NaCl, 0.1% Tween20) for 1 h at room temperature and incubated with primary antibodies overnight at 4 °C. Primary antibodies were detected using species-specific HRP-conjugated secondary antibodies. Blots were developed using Femto Maximum Sensitivity Substrate (Pierce) and imaged using a Protein Simple imaging system. Antibodies used included ARP3 (Cell Signaling Technology, #4738) PSD95 (NeuroMab clone K28/43, purchased from Biolegend #810401) and SynGAP (Cell Signaling Technology, clone D20C7, #5539), all at a 1:1000 dilution.

### Sample digestion

The dried samples were resolubilized in 40 µL 0.1% PPS Silent Surfactant (3-(4-(1,1-bis(hexyloxy)ethyl)pyridinium-1-yl)propane-1-sulfonate, a mass spectrometry compatible detergent) in 50 mM Tris pH 8. Disulfide bonds were reduced by addition of 0.8 µL 500 mM Tris (2-carboxyethyl) phosphine hydrochloride (TCEP) at 37 °C for 1 h, and the cysteines were subsequently alkylated using 1 µL of 500 mM iodoacetamide at room temperature for 30 min. Tryptic digestion proceeded for 5 h at 37 °C following the addition of 0.5 µg porcine trypsin (Pierce sequencing grade). Digestion was halted by addition of 2 µL 10% trifluoroacetic acid, and the PPS detergent was cleaved after 1 h at room temperature by this addition of acid.

### Liquid chromatography–tandem mass spectrometry (LCMS)

All mass spectrometry was performed on a Q-Exactive HF-X (Thermo Fisher Scientific) mass spectrometer with a Thermo Easy-nLC 1200 HPLC with autosampler. The dried tryptic peptides (~ 15 µg per sample) were solubilized in 40 µl of loading buffer. The loading buffer was comprised of 0.1% trifluoroacetic acid, 2% acetonitrile in water, and 10 fmol/µl of a peptide standard (Pierce PRTC). Sample volumes of 5 µl were injected via the autosampler onto a 150-μm Kasil fritted trap (Dr. Maisch Reprosil-Pur 120 C18-AQ 3 µm beads, 2 cm × 150 µm) at a flow rate of 2 µl/min. After loading and desalting using a total volume of 12 µl of loading buffer, the trap was brought on-line with a fritted packed tip (75-μm inner diameter, New Objective PicoFrit) packed to a length of 30 cm with the same Dr. Maisch beads. The column and trap were mounted to a nanospray ion source (CorSolutions, Ithaca, NY) heated to 50 °C, and placed in line with the HPLC pump. Peptides were eluted off the column using a gradient of 0–36% acetonitrile in 0.1% formic acid over 90 min, followed by 36–60% acetonitrile over 10 min at a flow rate of 250 nl/min.

The mass spectrometer was operated using electrospray ionization (2 kV) with the heated transfer tube at 300 °C, and data was acquired using data independent acquisition (DIA). For each sample, one MS1 spectrum (m/z 395–1005, 30,000 resolution) was acquired with every 75 targeted MS2 where the targeted m/z value was set to m/z 404.4337, and sequentially increased by m/z 8.0036 up to m/z 1000.704. After another MS1 scan, a new cycle of targeted MS2 scans was initiated where the center of each isolation window was staggered by m/z 4.0018 compared to the first round of MS2 (i.e., starting at m/z 400.4319, stepping up by m/z 8.0036 for each MS2, and ending with m/z 996.703). This pattern of data acquisition was repeated throughout each run (MS1, followed by 75 targeted MS2, followed by MS1, followed by targeted MS2 that had been offset by m/z 4). This staggered precursor range can be deconvolved^39^ resulting in mzML formatted files with an effective precursor isolation width of m/z 4. The use of non-integer targeted MS2 precursor values (i.e., m/z 404.4337 instead of m/z 404) allows for the quadrupole isolation edges to be at m/z values where doubly- and triply-charged tryptic peptide precursors are less likely to be observed. The HCD collision energy was set to 27%, and the precursor charge default state was set to two. The quadrupole isolation width was set to eight, and the MS2 resolution was 15,000. In addition to the analysis of individual samples, a pooled sample was analyzed in this manner at the beginning, middle, and end of the total acquisition period.

### DIA data analysis

Data analysis for the DIA data employed the computer program Encyclopedia v1.2.2^40^. A Prosit spectral library^41^ was created from a mouse FASTA file. Using a normal target/decoy approach, with both b- and y-ions, and a mass tolerance of 10 ppm, the runs from the pooled sample plus four other runs from wild type samples were analyzed together to make a chromatogram library. This chromatogram library includes information on the fragment ion m/z, relative intensity, and accurate retention times for all detected tryptic peptides in the pooled and wild type samples. This chromatogram library was then used by Encyclopedia to detect the presence of those peptides in all the individual samples using the wide isolation data acquisition scheme. The final step performed by Encyclopedia is to create the quantitative reports and one final library in which the chromatographic peak boundaries are set across all the runs, which ensures that each peptide is detected with roughly the same retention time. Further data manipulations and viewing was done within Skyline^42^ after importing this final Encyclopedia spectral library and the deconvolved mzML data files.

### Statistical analysis

− Log10pvalues and Log2 Fold Changes were computed in Microsoft Excel using a 2-tailed Student’s *t* test with no correction for multiple comparisons. Volcano plots were generated in Prism (GraphPad).

## Supplementary Information

Supplementary Information.

Supplementary Table S1.

Supplementary Table S2.

Supplementary Table S3.
