# Supplementary material for: Activity dependent dissociation of the Homer1 interactome
Source: Sci Rep. 2022 Feb 25;12:3207. doi: 10.1038/s41598-022-07179-3 (PMC8881602; doi:10.1038/s41598-022-07179-3)
Supplement: Supplementary file 1 — Supplementary Information. [file 41598_2022_7179_MOESM1_ESM.pdf]

Supplementary Table 1: A list of all proteins previously listed as Homer1-interacting proteins in BioGRID or Uniprot, indicating which proteins were identified in this study.

Supplementary Table 2: A list of all peptides identified by the mass spectrometer in the current study, showing p values and log2FoldChange values for each comparison group.

Supplementary Table 3: A list of all proteins identified by the mass spectrometer in the current study, showing p values and log2FoldChange values for each comparison group.

Supplementary Figure 1: Whole-blot images of the cropped western blots shown in Fig 4E.

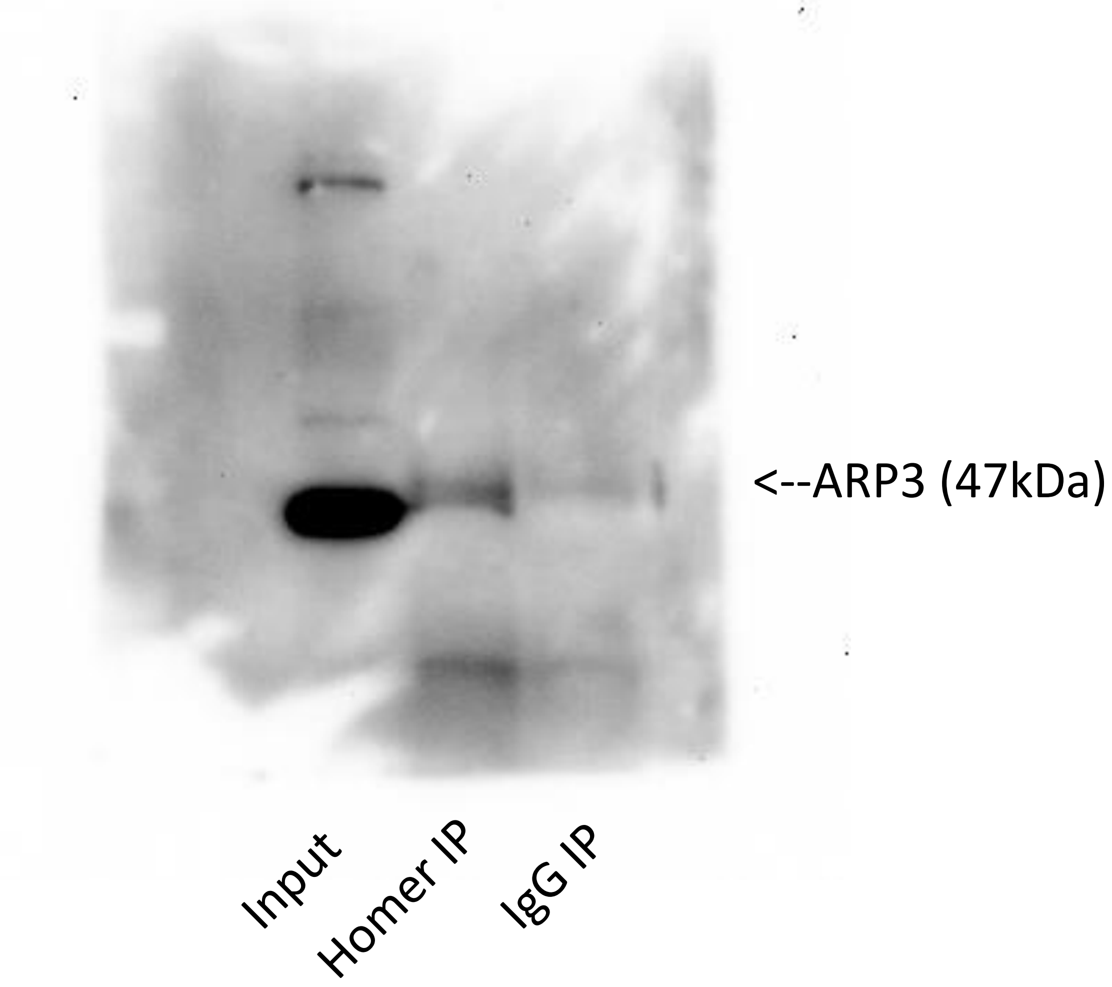

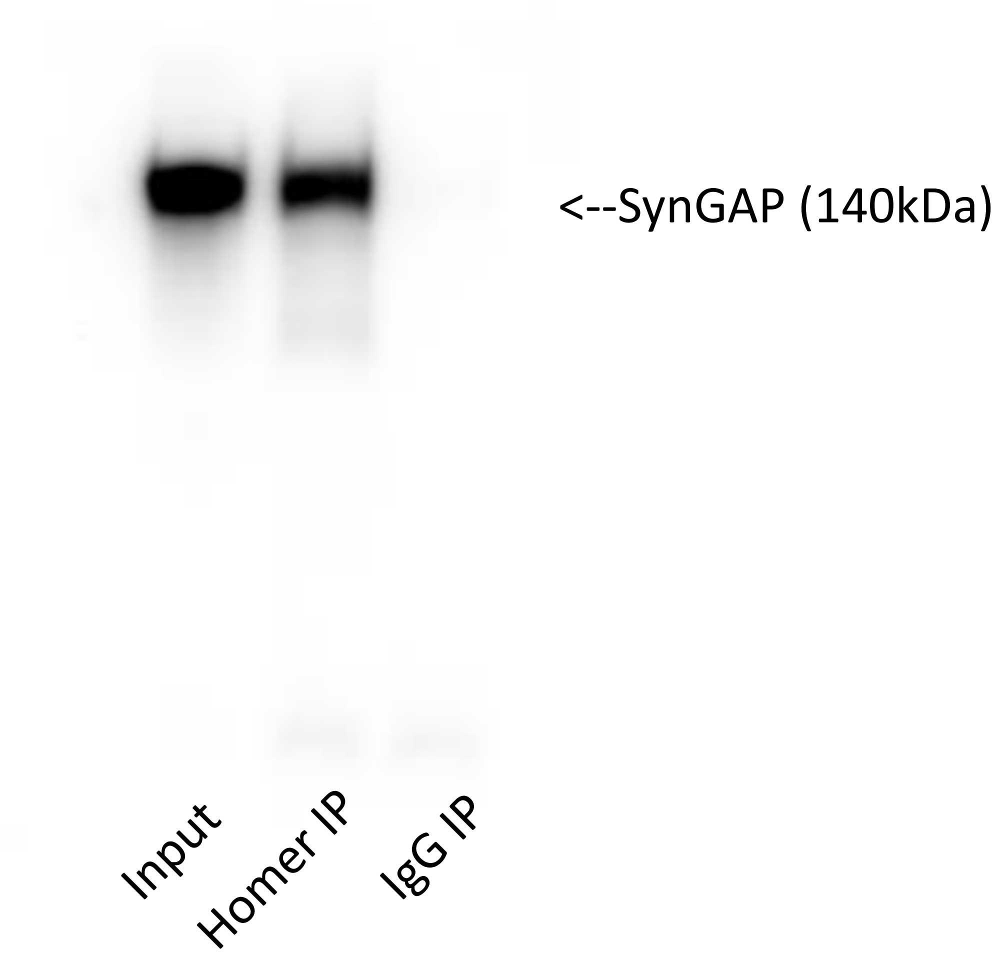
<--SynGAP (140kDa)
Input
Homer IP
IgG IP

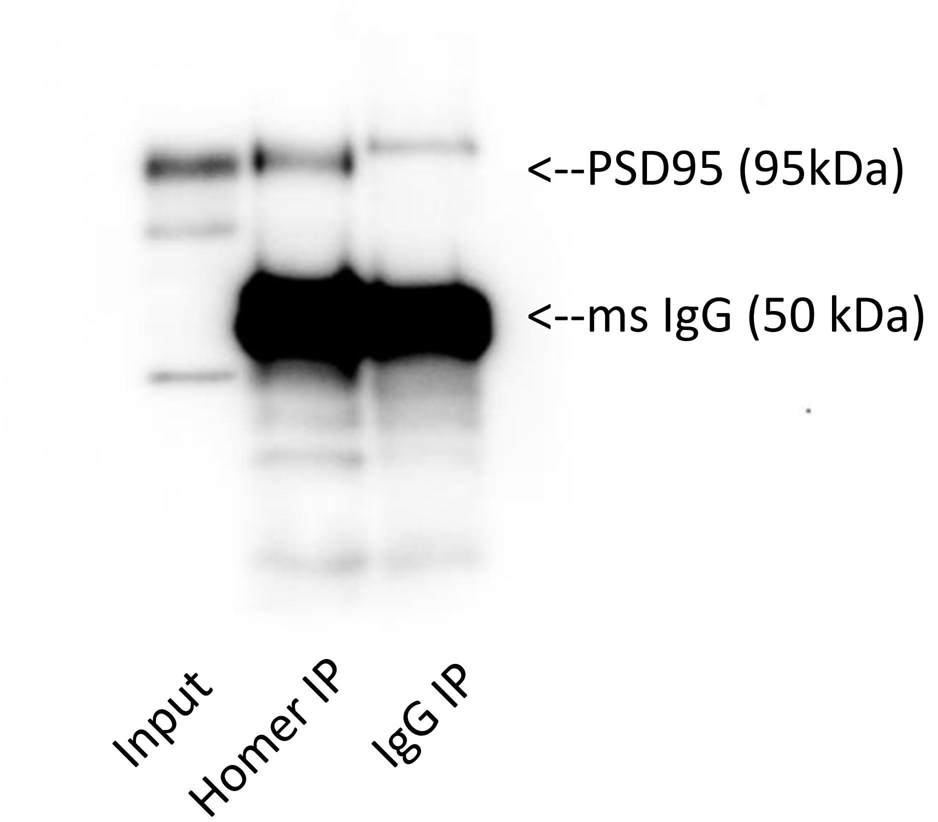

<--PSD95 (95kDa)
<--ms IgG (50 kDa)
Input
Homer IP
IgG IP

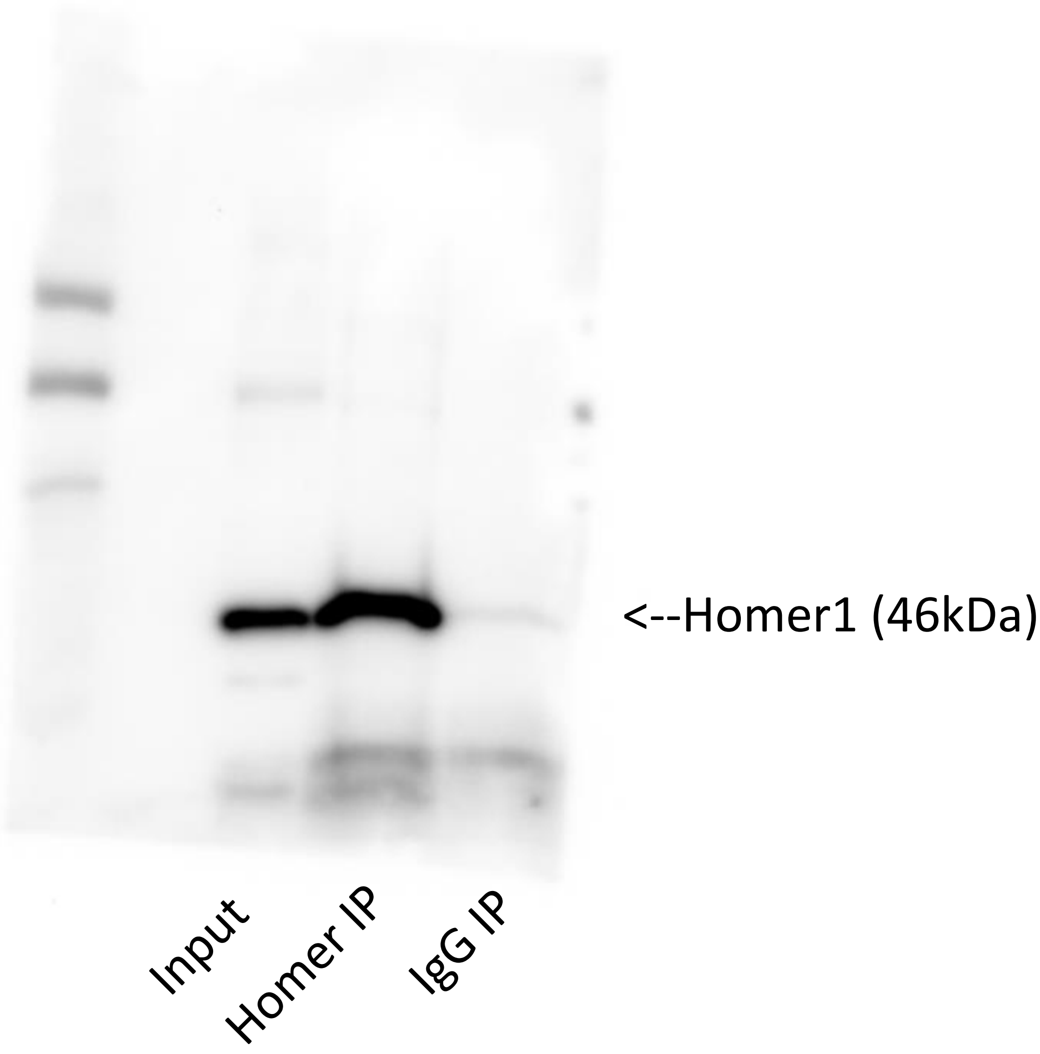

Input
Homer IP
IgG IP
<--Homer1 (46kDa)
